# Inflammation Biomarkers and Correlation to Wound Status After Full-Thickness Skin Grafting

**DOI:** 10.3389/fmed.2019.00159

**Published:** 2019-07-12

**Authors:** Karim Saleh, Ann-Charlotte Strömdahl, Kristian Riesbeck, Artur Schmidtchen

**Affiliations:** ^1^Division of Dermatology, Department of Clinical Sciences, Skane University Hospital, Lund University, Lund, Sweden; ^2^Clinical Microbiology, Department of Translational Medicine, Faculty of Medicine, Lund University, Malmö, Sweden; ^3^Department of Biomedical Sciences, Copenhagen Wound Healing Center, Bispebjerg Hospital, University of Copenhagen, Copenhagen, Denmark

**Keywords:** dermatologic surgery, wound healing, surgical site infection, full-thickness skin grafting, inflammation biomarkers

## Abstract

**Background:** A surgical site infection (SSI) is believed to be the result of an exaggerated inflammatory response.

**Objective:** Examine the relationship between clinical status and inflammation biomarkers in full-thickness skin grafting wounds.

**Methods:** Twenty patients planned for facial full-thickness skin grafting were enrolled. A week after surgery, all graft wounds were clinically assessed using a 3-step scale for inflammation (low, moderate, high). All wounds were swabbed for routine microbiological analysis and assessment of numbers of aerobic bacteria. Tie-over dressings from all patients were collected and used for wound fluid extraction and subsequent analysis of MMPs, cytokines, and NF-κB inducing activity.

**Results:** Wounds with a high degree of inflammation contained increased total MMP activity (*P* ≤ 0.05) in their corresponding fluids. Likewise, the level of the cytokines IL-1ß, IL-8, IL-6, TNF-α was analyzed, and particularly IL-1ß was discriminatory for highly inflamed wounds (*P* ≤ 0.01). Moreover, bacterial loads were increased in highly inflamed wounds compared to wounds with a low degree of inflammation (*P* ≤ 0.01). NF-κB activation in the monocytic cell line THP-1 was significantly higher when these cells were stimulated by wound fluids with a high degree of inflammation (*P* ≤ 0.01). Growth of *S. aureus* in wounds did not vary between wounds with different degrees of inflammation (chi-square 3.8, *P* = 0.144).

**Conclusion:** Biomarkers analyzed from tie-over dressings correlated to clinical wound healing in full-thickness skin grafting.

## Introduction

Surgical site infections (SSIs) are infections developing in surgical wounds within 30 days of surgery (1). They are the leading nosocomial infections in developing countries and the second most frequent nosocomial infections in Europe and the United States (2, 3). Within dermatologic surgery, the risk for an SSI to occur is assumed to be low and varying from 5 to 10% (4), but in skin-grafting procedures it can be as high as 28.5% (5). The pathogenesis for development of SSIs is complex and not fully understood. In normal conditions, acute wounds heal in a sequenced and timely manner, characterized by four major phases (namely hemostasis, inflammation, proliferation, and remodeling). This is a complex process involving chemokines, cytokines, proteases, and their respective counterregulatory molecules through the healing process (6). In normal skin, there is a healthy equilibrium between skin microbiota and innate immunity. During surgery, this equilibrium is disrupted due to tissue damage resulting in the start of a primed pre-triggered host immune-inflammatory process. Bacterial entry to wounds exacerbates this inflammatory response setting the wound in a non-healing state (7). The resulting inflammatory phase disrupts the balance between deposition and degradation of the extracellular matrix further inhibiting the wound healing (7).

Bacterial proliferation within a wound bed is also known to result in pathologic alterations to each phase of wound healing (8). For many years it has been thought a wound infection exists when the microbial load is <10^5^ colony-forming units (CFU) per gram of tissue (9). However, there is growing evidence suggesting the pathogenesis for an SSI is far more complex than dependent on just the bacterial load of a wound (10). It is today believed an SSI is the result of a primed inflammation to a surgical insult which is both dysregulated and dysfunctional due to the excessive immune-inflammatory response to invading pathogens in the postoperative phase (11).

To enhance our understanding of wounds, biomarkers have been studied widely in the last two decades. Biomarkers are defined as objectively quantifiable substances that can be assessed as an indicator of normal physiological and pathogenic processes, or pharmacologic responses to a therapeutic intervention (12). Investigations of different biomarkers in wound healing have mostly been based on studying chronic non-healing wounds (13). Of the various classes of biomarkers studied, proteases [particularly matrix metalloproteinases (MMPs)], protease inhibitors, and proinflammatory cytokines have the greatest potential in wound assessments (13). Chronic wounds have been shown to contain elevated levels of proinflammatory cytokines such as tumor necrosis factor-alpha (TNF-α) and the interleukins (IL) IL-1, IL-6, and IL-8 (14).

Wound fluid can easily be collected from a wound and is thought to give a relevant reflection of the wound status. It has therefore provided a valuable basis for wound research (15). Wound fluid consists mainly of a mixture of extravasated material and factors synthesized in the vicinity of the wound. Since wounds are colonized by a variety of bacteria, wound fluid represents a heterogeneous mixture of endogenously synthesized proteins from the underlying granulation tissue, bacterial products, and plasma proteins (16).

In this study, we aimed to analyse wound fluids collected from acute surgical wounds on the face after full-thickness skin grafting. Our objective was to examine different biomarkers and see if these correlated to the clinical status of wounds in terms of inflammation and healing. Another objective was to see if analyzed biomarkers were related to wound bacterial numbers and types.

## Materials and Methods

### Ethics Statement

The use of human wound materials was approved by the Ethics Committee at Lund University (2013/762). Informed consent was obtained from all patients enrolled.

### Patients and Biological Samples

**Figure 1:**
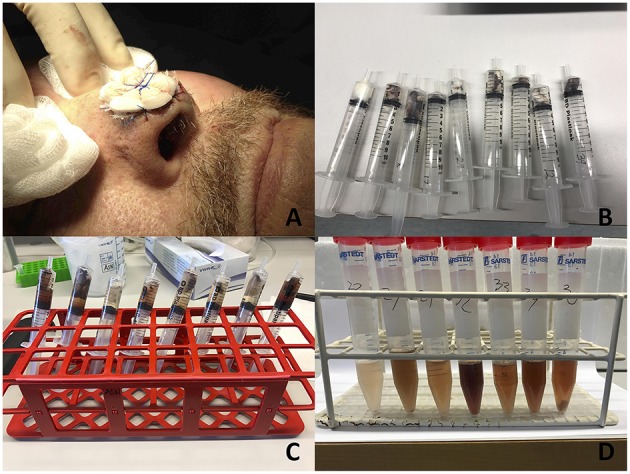
Illustration showing how wound fluids were collected. **(A)** Illustration of a tie-over dressing sutured on top of a skin transplant. Written informed consent was obtained from the participant for the publication of this image. The tie over dressings were collected a week after surgery from all patients. **(B)** Tie over dressings placed in sterile syringes. **(C)** Extraction medium (Tris buffer) added to syringes which were put on a shaker for 1 h. **(D)** Wound fluid collected after squeezing out the contents of each syringe to a sterile tube).

Wound fluids have been extracted from wound dressings in previous studies (17). Wound fluids in this study were extracted from tie-over dressings belonging to 20 patients that underwent facial full-thickness skin grafting at our clinic and constituted the control group in a randomized controlled trial we previously published (18). A tie-over dressing is a dressing sutured on top of the skin transplant and kept in place for a week after surgery to protect the transplant and to prevent hematoma formation (Figure 1A). All tie-over dressings obtained were cut from Mepilex® (Mölnlycke Healthcare, Göteborg, Sweden), an absorbent polyurethane foam dressing. None of the patients received antibiotic treatment. There were 7 males and 13 females, with a median age of 85 years (range 45–91 years). A week after surgery, tie-over dressings were collected and immediately stored at −20°C for later wound fluid extraction. Digital photographs were taken of all wounds after obtaining informed consent.

At the day of extraction, all dressings were placed into sterile syringes (Figure 1B). Extraction medium consisting of 10 mM Tris buffer (pH 7.4) was added to each syringe. Minimum amount of medium in each syringe was 3.5 ml and then an additional 2 μl/mg of the weight of the dressings was added to adjust for different dressing weights (Figure 1C). All syringes were sealed and then put onto a shaker in 8°C for 1 h. Thereafter, each syringe was emptied into a sterile 5 ml Falcon tube (Figure 1D) by pressing the plunger firmly down the barrel, flushing all liquid through the dressing. Extracted fluids were then aliquoted in 200 μl samples for later downstream analyses. Protein concentration in each sample was determined by the BCA assay (Thermo Fischer Scientific, Hvidovre, Denmark).

### Sodium Dodecyl Sulfate Polyacrylamide Gel Electrophoresis (SDS-PAGE)

SDS-PAGE was performed as described by Laemmli (1970) using Novex® pre-cast gel system (Invitrogen, Carlsbad, CA) on Tricine gels with a 4% stacking gel (Invitrogen). Pierce™ protease and phosphate inhibitor (Thermo Fischer Scientific) was added to extracted wound fluids. Next, wound fluid samples amounting to 20 μg protein were denatured at 85 °C for 4 min in 2x Tricine SDS sample buffer and 10x NuPAGE® reducing agent followed by separation on 10–20% Tricine gels in 1x Tricine SDS running buffer for 60 min at 125 V. After fixing, the gels were stained with Coomassie brilliant blue, destained, and protein patterns were visualized using Image Lab software (Bio-Rad Laboratories, Hercules, CA).

### Zymography

Wound fluids derived from the dressings (5 μg total protein) were mixed with sample buffer (0.4 M Tris HCl, 20% glycerol, 5% SDS, 0.03% bromophenol blue, pH 6.8) and separated on 10% polyacrylamide gels (1 mg of bovine gelatin per ml of gel). To remove SDS, gels were incubated with 2.5% Triton X-100. Incubation was then performed for 18 h at 37°C in buffer containing 50 mM TrisHCl, 200 mM NaCl, 5 mM CaCl_2_, 1 μM ZnCl_2_, pH 7.5. Gels were stained with Coomassie blue G-250 in 30% methanol, 10% acetic acid for 1 h, and destained in the same solution without the dye. Gelatinase-containing bands were visualized as clear zones against a dark background using an Agfa Duoscan 1,200 in the transillumination mode. Three identical zymography experiments were performed. Pictures were processed using Image Lab software (Bio-Rad Laboratories) for determining band intensities representing the sum of all detected MMPs (MMP-2, 9, MMP-complex) for each sample.

### Azocasein Assay

Total protease activity from each wound fluid was determined by the azocasein method as described by Tomarelli (19). Hundred μg of total protein from each wound fluid sample were added to 50 μl azocasein substrate (2% azocasein enzyme substrate in 10 mM Tris HCl, 8 mM CaCl_2_, pH 7.4). The reaction mixture was incubated in 37 °C for 24 h. Thereafter, 240 ml 10% trichloroacetic acid was added and the samples mixed and allowed to stand for 15 min to ensure complete precipitation of undigested material. Tubes were centrifuged at 10,000 rpm (microfuge) for 5 min and 125 μl of the supernatant was transferred to wells of a 96-wells plate containing 125 μl 1.0 M NaOH. The absorbance at 440 nm was determined. Results given represent mean values from triplicate measurements.

### NF-κB Activity Measurement

NF-κB was assessed in the transfected THP1-Xblue™-CD14 reporter cell line (hereafter denoted THP1-reporter cells) (InvivoGen, San Diego, CA) according to the manufacturer's instructions. Briefly, cells were grown in RPMI 1,640 with 10% (v/v) heat-inactivated FBS, 1% Antibiotic–Antimycotic (Invitrogen), 100 μg/ml G418, and 200 μg/ml of Zeocin. Cells were centrifuged at 250 × g for 5 min and resuspended at 1.3 × 10^6^ cells/ml in RPMI supplemented with 10% heat-inactivated FBS and 1% Antibiotic–Antimycotic solution. Subsequently, 180 μl cell suspension was added to a 96-well plate. THP-1 cells were thereafter stimulated with the wound fluid derived from the dressings (50 μg protein each). *Escherichia coli* LPS (1 μg/ml) was used as a positive control. After incubation (18–22 h) at 37°C and 5% CO_2_, enzyme activity was determined in 20 μl of supernatant by using 180 μl QUANTI-Blue substrate (InvivoGen). Plates were incubated at 37°C and the level of secreted embryonic alkaline phosphatase (SEAP), an indicator of activation of transcription factor NF-κB, was measured after 1–2 h at OD 600 nm. Results given represent mean values from triplicate measurements.

### Cytokine Assay

Cytokine levels in the wound fluids were determined by measuring IL-1ß, IL-6, IL-8, and TNF-α concentrations, using ELISA kits (R&D Systems, Minneapolis, MN) according to the manufacturer's instructions. Ten μl of wound fluid from each patient was used for the assay. Results given represent mean values from triplicate measurements. Cytokine levels were expressed as concentrations relative to the surface area of the transplanted wound (**Figure 4A**) and total wound fluid protein (**Figure 4C**), respectively.

### Clinical Assessments of Wounds

**Figure 2:**
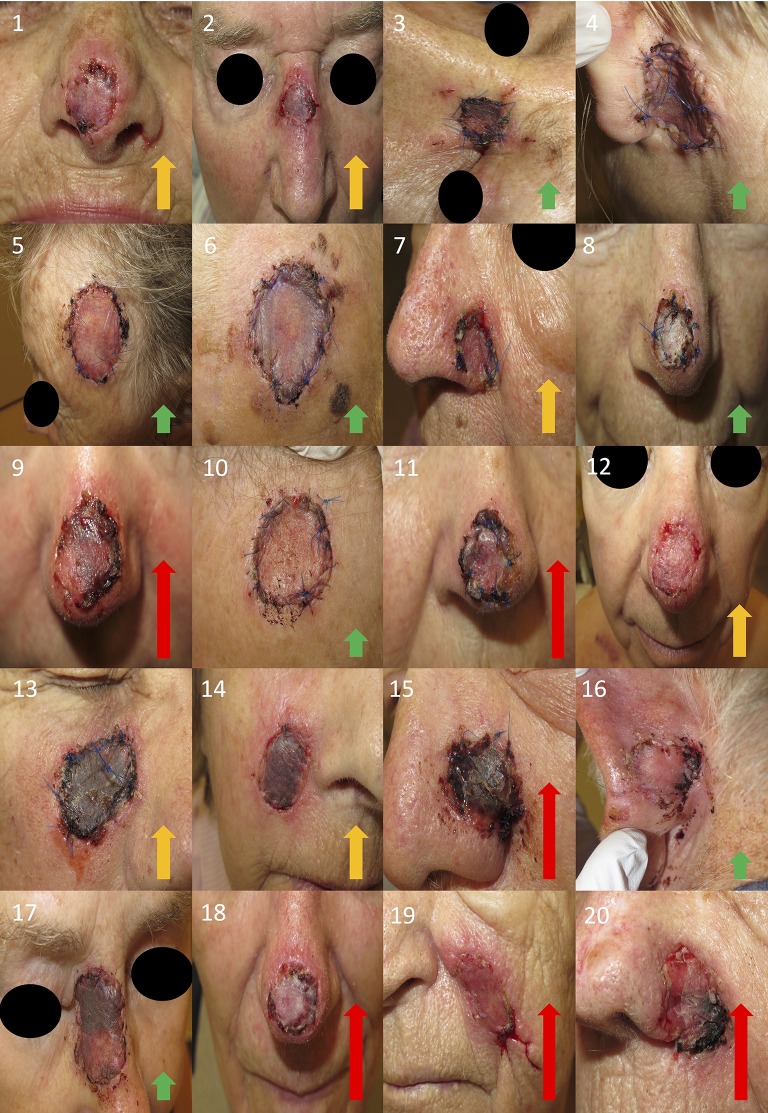
Photographs of all wounds. Written informed consent was obtained from all the participants for the publication of these images. Wound inflammation was assessed as mild (green arrow), moderate (orange arrow), or high (red arrow).

Each wound photograph was blindly assessed by two independent examinators (KS and AS) in terms of visual criteria using a three-step scale (low, moderate, high) for degree of inflammation (Figure 2). This was not a validated method of assessment.

### Microbiota

After each tie-over dressing was removed, all wounds were swabbed. Each swab was analyzed quantitatively by counting colony forming units (CFU) per cm^2^ of area swabbed as well as the type of bacteria present. Bacterial quantification was done by serially diluting each swab to 3 different concentrations plating each concentrate onto a Todd-Hewitt agar plate using sterile glass beads and incubating all plates in 5% CO_2_ at 37°C for 24 h. CFU were thereafter counted, and between 30 and 300 CFUs were observed on plates. CFU values obtained were divided with the swab area to measure bacterial loads in CFU/cm^2^. Bacterial species were determined via matrix-assisted laser desorption/ionization time-of-flight (MALDI-TOF) mass spectrometry. Isolates were prepared for acquisition of spectra using a standard ethanol-formic acid extraction protocol developed by the mass spectrometer manufacturer (Bruker Daltonics, Bremen, Germany). The bacterial pellets were allowed to dry completely (up to 1 h) following ethanol washing. The volume of formic acid anacetonitrile used (10–40 μl) was based on pellet size. Mass spectra of isolates were acquired using a microflex MALDI-TOF mass spectrometer with flexControl software (Bruker Daltonics, Bremen, Germany) using default settings (mass range of spectra, m/z 2,000 to 20,000 in linear positive-ionization mode). All bacteria identified after plating were recorded. Confirmatory assays included slide agglutination test (Pastorex, Bio-rad) for verification of *S. aureus*. No fungal species were detected in these analyses. Cultures for anaerobes were not performed as this would optimally require biopsies and separate microbiological analyses which were not possible in the present clinical setting. Data on bacterial species and CFU numbers for this patient group have been included as controls in a previously published study on effects of antiseptics (18).

### Statistics

Results are shown as mean values with SEM. To compare more than two groups, one-way ANOVA with the Kruskal-Wallis test was used. Differences in categorical variables were determined using chi-square test. All statistical evaluations were performed using GraphPad Prism software 7.0 with ns, not significant, ^*^P ≤ 0.05 and ^**^P ≤ 0.01.

## Results

Tie-over dressings from 20 wounds belonging to patients that underwent facial reconstruction by full-thickness skin grafting were collected 1 week after surgery. Wound fluids were extracted from all tie-over dressings followed by determination of protein content (Figure 1). The wounds exhibited variable degrees of inflammation. Inflammation was evaluated by 2 independent examinators using a 3-step scale (low, moderate, and high) and the overall estimate was presented (Figure 2).

**Figure 3:**
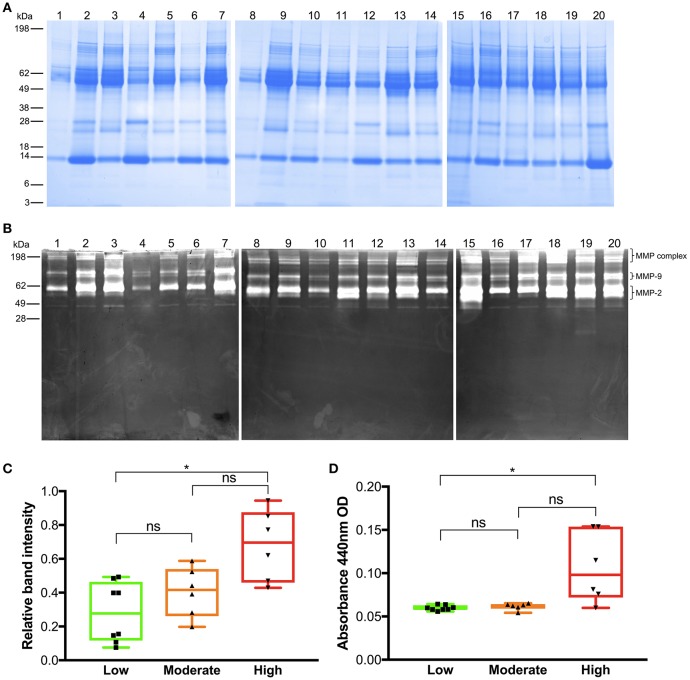
Analysis of wound fluids. **(A)** SDS-PAGE analysis of all wound fluid extracts using 20 μg protein from each sample. Numbers on the left indicate molecular weights for the standard proteins in kDa. Wound fluid sample numbers are indicated on the top (images of one representative experiment are shown, *n* = 3, three separate gel images are aligned next to each other). **(B)** Gelatin zymography analysis of wound fluids. Each lane was loaded with 5 μg of total protein from each sample. Numbers on the left indicate molecular weights for the standard proteins in kDa. Wound fluid sample numbers are indicated on the top (image of one representative experiment is shown, *n* = 3, three separate gel images are aligned next to each other). Brackets illustrate positions of the respective MMP classes. **(C)** Relative band intensity of MMP-2,−9, and MMP complexes on gelatin zymograms of all wound fluid samples was analyzed using Image lab software. Patients with high inflammation showed higher levels of MMP activity. **(D)** Azocasein assays illustrating total protease activity of all wound fluid samples according to degree of inflammation (Kruskal-Wallis test; ns, not significant; *P ≤ 0.05).

In order to illustrate the overall protein composition, all wound fluids were first analyzed by SDS-PAGE (Figure 3A). The results showed similar patterns, with bands ranging from low molecular proteins of 10 kDa, up to higher molecular weight proteins of 100–200 kDa. A prominent band of about 60 kDa, corresponding to human albumin was observed. Overall, the patterns were similar to previous results on collected wound fluids (16, 20), thus validating the present methodology. Zymography is a widely used method for studying of proteinase activities during wound healing, employed in studies on both acute and non-healing ulcers (21–23). Equal amounts of proteins from all wound fluids were separated on 10% gelatin gels, and the presence of proteases visualized (Figure 3B). Bands at ~70 and 100 kDa corresponded to those previously identified as MMP-2 and−9, respectively (23). Notably, double bands were noted, corresponding to the pro-forms and active MMPs (24). Differences in band intensities were detected in wound extracts from the different patients. In order to analyse the total activity of the wound fluids in a semiquantitative way, the resulting zymograms were analyzed for total band intensity. We decided to analyse total MMP activity, corresponding to MMP-2,−9, and the MMP complex (25). Wound fluids from wounds with a high degree of inflammation displayed significantly higher MMP levels than wounds with moderate or low degrees of inflammation (Figure 3C). This correlated very well with total protease activity, as assessed by an azocasein assay (19), which demonstrated significantly higher azocasein-degrading activity in wound fluid material derived from wounds with a high degree of inflammation compared to wounds with low or moderate inflammation (Figure 3D).

**Figure 4:**
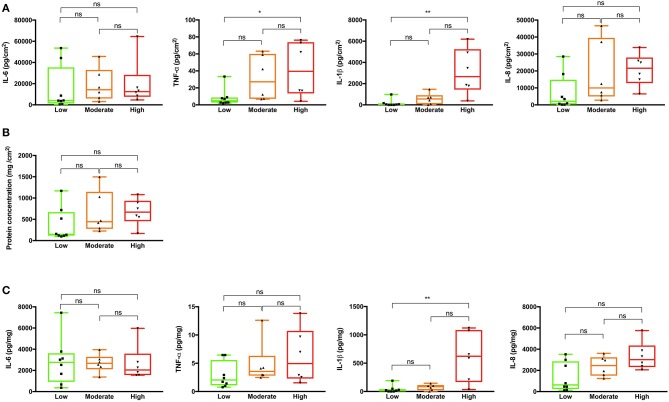
Cytokine and protein analyses of wound fluids extracted from tie-over dressings. **(A)** The cytokines IL-6, TNF-α, IL-1ß, and IL-8 were measured in the wound fluids derived from different patients. The total cytokine load per cm^2^ of the dressing area is indicated on the y-axis. **(B)** Protein concentrations in the respective dressings extracts were determined using a BCA assay. The protein content per cm^2^ of the dressing area is indicated on the y-axis. **(C)** The concentrations of the cytokines IL-6, TNF-α, IL-1ß, and IL-8 in the dressings extracts are presented on the y-axes. In **(A–C)** Kruskal-Wallis test was used; ns, not significant; *P ≤ 0.05; **P ≤ 0.01.

Next, we explored the levels of proinflammatory cytokines in the different wound fluids. The results showed that TNF-α and IL-1ß (Figure 4A), normalized relative to the respective wound surface areas, were significantly higher in wounds with more pronounced inflammation, when compared to wounds with a low degree of inflammation. In particular, IL-1ß appeared to show a high specificity, with significantly increased levels in wounds graded as highly inflamed, and with low recorded levels in the groups with low and moderate inflammation (Figure 4A). As wound inflammation may be correlated to total protein leakage, absorbed into the dressing, we next evaluated the relationship between the protein concentration of the dressing extracts and surface area. The results showed no statistically significant differences, although a trend was observed with increased protein content, when normalized to wound area, with higher inflammation grade (Figure 4B). Finally, when presenting the cytokine concentration relative the total protein content, only IL-1ß showed a significant increase in the wounds with high inflammation, although a trend was observed for particularly IL-8 (Figure 4C).

**Figure 5:**
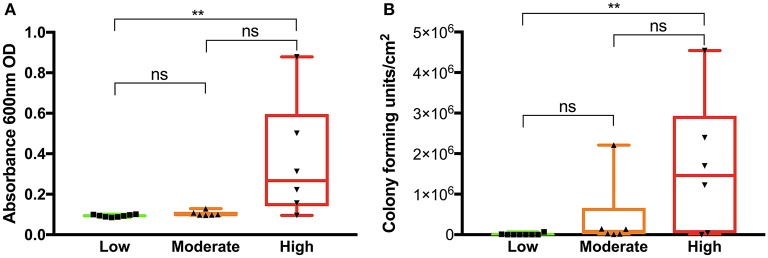
Analysis of pro-inflammatory activity of wound fluids and determination of bacterial levels. **(A)** THP1-Blue NF-κB cells were incubated with wound fluid extracts (50 μg total protein each) and activation of NF-κB was measured. Wound fluids from patients having a high degree of inflammation showed an increased NF-κB activation in the THP-1 cell line. **(B)** The wound areas were swabbed and bacterial CFU were determined after plating. Wounds showing high inflammatory signs contained higher bacterial levels. In **(A,B)** Kruskal-Wallis test was used; ns, not significant; **P ≤ 0.01.

In a reductionistic approach, we next measured the impact of the wound fluids on inflammation *in vitro* by determining their NF-κB activating capacity on THP-1 monocytic reporter cells *in vitro*. The results showed that particularly fluids from wounds with high inflammation were able to induce NF-κB activation (Figure 5A). The wounds with a high degree of inflammation also contained higher loads of bacteria grown under aerobic conditions, as determined by measuring the CFU derived from the swabs of the respective wound surfaces (Figure 5B). Bacterial species isolated from all wounds 1 week after surgery varied significantly (Table 1). The most common species was *S. epidermidis*, and we found *S. aureus* being the second most common species in a total of 7 wounds. The presence of *S. aureus* in wounds did not vary between wounds with different degrees of inflammation (chi-square 3.8, *P* = 0.144), albeit a tendency of *S. aureus* presence was associated with high inflammation.

**Table 1:** The table shows bacterial species isolated from the wounds, bacterial numbers, and degrees of wound inflammation.

**Patient**	**Species**	**CFU/cm^**2**^**	**Inflammation degree**
1	*S. aureus*	1.4 × 10^5^	Moderate
2	*S. epidermidis* and *S. pasteuri*	1.4 × 10^5^	Moderate
3	*S. aureus*	6.5 × 10^2^	Low
4	*S. epidermidis*	6.8 × 10^2^	Low
5	No growth	0	Low
6	*S. aureus* and *S. hominis*	0	Low
7	*S. epidermidis* and *S. capitis*	1.8 × 10^4^	Moderate
8	*S. epidermidis*	1 × 10^4^	Low
9	*S. epidermidis*	4.5 × 10^4^	High
10	*S. epidermidis*	0	Low
11	*S. aureus*	4.5 × 10^6^	High
12	*S. epidermidis*	2.9 × 10^4^	Moderate
13	*S. epidermidis*	1.1 × 10^4^	Moderate
14	*S. epidermidis*	2.2 × 10^6^	Moderate
15	*S. aureus*	1.3 × 10^7^	High
16	*S. capitis*	1 × 10^3^	Low
17	*S. epidermidis*	0	Low
18	*S. aureus*	1.2 × 10^6^	High
19	*S. epidermidis* and *S. capitis*	6.6 × 10^2^	High
20	*S. aureus* and *S. epidermidis*	1.7 × 10^6^	High

*Samples were collected one week after surgery*.

## Discussion

To the best of our knowledge, this is the first study that examines wound fluids extracted from tie-over dressings from wounds covered with full-thickness skin grafts. Skin grafting surgery is normally associated with a higher risk of SSI (5) which is why we decided to examine wounds from this type of dermatologic surgery. We found that wounds that had a clinical appearance of being more inflamed indeed had higher levels of proteases, as demonstrated for the major MMPs, MMP-2 and−9. These wounds had increased cytokine levels relative to the wound surface area, particularly observed for IL-1ß, but also for TNF-α. Wound fluids from highly inflamed wounds yielded significantly higher NF-κB activation in THP-1 reporter cells. Moreover, these wounds also had significantly higher overall bacterial loads, a factor thought to have importance in the development of SSIs, an observation that we previously published (26). It is therefore plausible that an increased bacterial burden in acute wounds leads to increased levels of proinflammatory cytokines, as has previously been shown for chronically infected leg ulcers (27), thus increasing the overall risk for wound complications and eventually SSI development during acute wound healing. Being a protein complex that is a key regulator for the inflammatory response, NF-κB is known to play a crucial role in the wound healing process (28). An overexpression of NF-κB is thought to be associated with impaired wound healing (29). Taken together, our observation demonstrating that wound fluids from wounds showing high inflammatory signs indeed induce NF-κB activation in monocytic cells *in vitro*, could therefore provide a link between excessive inflammation, bacterial presence, and a hampered healing of surgical wounds.

While a tightly controlled expression of MMPs is a critical part of normal wound healing, elevated and prolonged expression can lead to excessive extracellular matrix degradation associated with impaired healing (7). Previous studies have shown that various proteases could serve as biomarkers of poor healing (22, 30, 31). It was therefore relevant that zymographic analysis showed that MMPs were increased in those wounds that were judged to have high inflammation, and thus at risk of poor wound healing. Previously, the azocasein assay has been used to detect levels of MMPs in studies on proteases in chronic wounds (21). Using the azocasein assay in the current study setting of acute wound healing, we were able to corroborate the findings obtained with zymography, showing an association between elevated protease activity and a high degree of wound inflammation.

It was notable that IL-1β was particularly detected in highly inflamed wounds. Secretion of IL-1β is regulated in a two-step process. Firstly, stimulation through among others toll-like receptors (TLR) induces their synthesis as inactive precursors that lack a signal peptide. Secondly, inflammasome activation catalyzes the posttranslational processing that is required for their secretion and bioactivity. It should also be noted that although IL-1β processing is catalyzed mainly by caspase-1, other proteases can also process IL-1β under particular circumstances, like during high neutrophilic inflammation (32), indeed compatible with the present findings on highly inflamed surgical wounds.

In a recent study, it was found that early collection of wound fluids and analysis of IL-1β and TNF-α could predict clinical outcome regarding SSIs after neck dissection surgery (33), further illustrating the potential of using cytokines such as IL-1β and TNF-α as specific biomarkers for wound healing. The method used here, based on extraction of tie-over dressings to obtain wound fluid material, proved to be a simple and painless way to examine wound biomarkers, which could potentially act as “reporters” for wound healing outcome and SSIs. In this context, it is worth mentioning that wound fluids have shown superiority over serologic markers in assessing ulcer inflammation in chronic wounds (27).

Whether the here reported method based on cytokine detection will reach a sufficient sensitivity and specificity in order to be able to be of diagnostic use has to be evaluated in future clinical studies on a larger scale. For example, although an assay for assessment of inflammation in non-healing leg ulcers, Woundchek™ was shown to have a good sensitivity for detection of neutrophil elastase and MMP levels, the predictive potential of protease measurements, although showing promising results (34), needs further clinical studies. Hypothetically, combining the use of markers such as IL-1β with analyses of MMPs and bacterial antigens could yield higher diagnostic sensitivity and specificity with respect to wound status, enabling a simultaneous determination of bacteria, host proteolytic activities, as well as selected cytokines. Indeed, ongoing research on biosensors which can be incorporated into dressings could make future integration of such capabilities in clinical use possible (35). The levels of biomarkers such as the cytokines determined here were therefore calculated relative to the surface area as well as total protein content, and thus of high relevance for development of sensors measuring total biomarker load or concentration, respectively.

Another study objective was to examine the association of bacterial species to wound healing and to the biomarkers analyzed. Although statistically not significant (*P* = 0.144), the presence of *S. aureus* in wounds postoperatively appeared to be associated with a high degree of wound inflammation and hence increased biomarker levels. A larger sample size would have been necessary to try to establish a significant association. It should be noted that anaerobes were not specifically cultured in this study. In clinical practice, the predominant *S. aureus*, and occasionally ß-hemolytic streptococci are the primary causes of infection in acute wounds of various types (36). Analogously, during full thickness grafting procedures, *S. aureus* was found in approximately 80% of the infected surgical wounds (26), motivating the focus on aerobic bacteria and clinically standardized and fast methods such as quantitative swab culture (37) followed by Maldi-TOF techniques for bacterial detection. It is possible, however, that anaerobes, such as *Bacteroides* and *Peptostreptococcus* spp. (36) also contribute to the inflammation observed in the full thickness transplants studied here. Analyses of these and other species would optimally require biopsies in combination with separate microbiological analyses, possibly in combination with DNA and RNA based detection methods (38, 39) and next generation sequencing (NGS) (40). However, as the present proof-of-concept study focused on inflammation biomarkers, such as MMPs and cytokines, and their respective correlation to clinical wound status, such extensive microbiome analyses were outside the scope of this clinically oriented study, and will be the impetus for future investigations.

A further limitation of this study was the subjective assessment of wounds in terms of inflammation. However, this is the reality when surgical wounds are assessed by clinicians, and was performed according to regular clinical routines. For example, when diagnosing SSIs, clinicians rely on subjective wound characteristics such as erythema, oedema, heat, and secretion. Studies show both intra- and inter-observer variations in assessing SSIs (41). According to most common definition used for SSIs (1), a wound can be assessed as infected solely on the clinician's subjective interpretation. Objective tools that measure wound parameters are currently lacking. Even though we relied on a visual assessment of inflammation degrees, our assessment appeared to provide a fair wound status considering these degrees matched different levels of bacterial loads. Hence, if the wounds had been grouped according to low, moderate, and high bacterial levels, the same significant results for the biomarkers would have been obtained. A wound with a higher bacterial load has higher levels of biomarkers and appears to be more inflamed visually.

In conclusion, we show that dressings can be used to collect wound fluids in the setting of full-thickness skin grafting. Biomarkers analyzed, such as MMPs and IL-1β, correlated to wound status. It remains to be evaluated at which stage biomarkers need to be obtained in order to predict development of SSIs. The biomarkers used in this study, and in particular, in combination with our method employing tie-over dressings, could potentially be used to monitor wounds bed side and evaluate different therapeutic approaches. Moreover, these data could provide valuable input for future development of sensitive and specific biosensors, enabling rapid evaluation of infection-inflammation, and wound healing. Ultimately, there is a huge need to improve surgical outcomes in all types of surgery in the future.

## Ethics Statement

Ethics approval was granted by the Ethics Committee in Malmö/Lund, registration number (2013/762).

## Author Contributions

KS and AS: conception and design. KS, A-CS, KR, and AS: development of methodology, analysis, interpretation of data, writing, review, and revision of the manuscript. KS and A-CS: acquisition of data. AS: study supervision.

### Conflict of Interest Statement

The authors declare that the research was conducted in the absence of any commercial or financial relationships that could be construed as a potential conflict of interest.
